# Supplementary material for: Exploring Genetic Diversity and Population Structure of Australian Passion Fruit Germplasm
Source: BioTech (Basel). 2025 May 16;14(2):37. doi: 10.3390/biotech14020037 (PMC12101393; doi:10.3390/biotech14020037)
Supplement: Supplementary file 1 [file biotech-14-00037-s001.zip › biotech-3571705-supplementary.pdf]

Supplementary Materials: Exploring Genetic Diversity and Population Structure of Australian Passion Fruit Germplasm

Xinhang Sun, Peter Bundock, Patrick Mason, Pragya Dhakal Poudel, Rajeev Varshney, Bruce Topp and Mobashwer Alam

Table S1. List of the 94 rootstock accessions used in the genetic diversity study.

ID	Origin	ID	Origin
17-001_A	McGuffies Red	17-016_C	Bunnings Pandora x QPDI.f
17-001_B	McGuffies Red	17-016_D	Bunnings Pandora x QPDI.f
17-001_C	McGuffies Red	17-017_A	McGuffies red x Bunnings Pandora
17-001_D	McGuffies Red	17-017_B	McGuffies red x Bunnings Pandora
17-002_A	McLeod pandora	17-017_C	McGuffies red x Bunnings Pandora
17-002_B	McLeod pandora	17-017_D	McGuffies red x Bunnings Pandora
17-002_C	McLeod pandora	17-018_A	McGuffies red x McLeod Pandora
17-002_D	McLeod pandora	17-018_B	McGuffies red x McLeod Pandora
17-003_B	McGuffies red x McLeod pandora	17-018_C	McGuffies red x McLeod Pandora
17-003_C	McGuffies red x McLeod pandora	17-018_D	McGuffies red x McLeod Pandora
17-003_D	McGuffies red x McLeod pandora	17-019_A	DPI P. flavicarpa
17-004_A	McLeod Pandora x McGuffies red	17-019_B	DPI P. flavicarpa
17-004_B	McLeod Pandora x McGuffies red	17-019_C	DPI P. flavicarpa
17-004_C	McLeod Pandora x McGuffies red	17-026_A	Heuston
17-004_D	McLeod Pandora x McGuffies red	17-026_C	Heuston
17-005_A	Bunnings Pandora x McGuffies red	17-026_D	Heuston
17-005_B	Bunnings Pandora x McGuffies red	17-027_A	McLeod rootstock
17-005_C	Bunnings Pandora x McGuffies red	17-029_A	Pandora
17-005_D	Bunnings Pandora x McGuffies red	17-029_B	Pandora
17-007_A	McGuffies red	17-029_C	Pandora
17-007_B	McGuffies red	17-029_D	Pandora
17-007_C	McGuffies red	17-264_A	McLeod x Bunnings Pandora
17-007_D	McGuffies red	17-264_B	McLeod x Bunnings Pandora
17-008_A	Q.DPI.f	17-264_C	McLeod x Bunnings Pandora
17-008_B	Q.DPI.f	17-264_D	McLeod x Bunnings Pandora
17-008_C	Q.DPI.f	17-265_A	DPI x McGuffies Red
17-008_D	Q.DPI.f	17-265_B	DPI x McGuffies Red
17-009_A	McGuffies red x Griffiths pandora	17-265_C	DPI x McGuffies Red
17-009_B	McGuffies red x Griffiths pandora	17-265_D	DPI x McGuffies Red
17-009_C	McGuffies red x Griffiths pandora	17-266_A	McLeod Pandora x DPI
17-009_D	McGuffies red x Griffiths pandora	17-266_B	McLeod Pandora x DPI
17-010_A	Q.DPI.f x Bunnings Pandora	17-266_C	McLeod Pandora x DPI
17-011_A	Griffiths Pandora	17-266_D	McLeod Pandora x DPI
17-011_B	Griffiths Pandora	17-267_A	Griffiths x McLeod
17-011_C	Griffiths Pandora	17-267_B	Griffiths x McLeod

17-011_D	Griffiths Pandora	17-267_C	Griffiths x McLeod
17-012_A	Q.DPI.f x McLeod Pandora	17-267_D	Griffiths x McLeod
17-012_B	Q.DPI.f x McLeod Pandora	17-268_A	Bunnings x Griffiths Pandora
17-012_C	Q.DPI.f x McLeod Pandora	17-268_B	Bunnings x Griffiths Pandora
17-012_D	Q.DPI.f x McLeod Pandora	17-268_C	Bunnings x Griffiths Pandora
17-016_A	Bunnings Pandora x QPDI.f	17-268_D	Bunnings x Griffiths Pandora
17-016_B	Bunnings Pandora x QPDI.f	17-269_A	Griffiths x Bunnings
SP16-22D	Sweetheart x Pandora	SP16-78	Sweetheart x Pandora
SP16-36D	Sweetheart x Pandora	SP16-90	Sweetheart x Pandora
SP16-42D	Sweetheart x Pandora	SP16-91	Sweetheart x Pandora
SP16-6	Sweetheart x Pandora	Dur_McL_Pandora	McLeod Pandora
Dur_Grif_ Pandora	Griffiths Pandora	Dur_Bun_Pandora	Bunnings Pandora

Table S2. List of the 95 scion accessions used in the genetic diversity study.

ID	Origin	ID	Origin
S1	295_R10-15	S49	96A_R5_2
S2	295_R10-5	S50	96A_No12_R4_14
S3	295_R10-8	S51	96A_No12_R4_15
S4	296_R10-10	S52	96A_No12_R6_8
S5	302_R10-2	S53	96A_No12_R6_15
S6	AG27_R5-10	S54	DB_1_R5_16
S7	AT-1_R17-8	S55	DPI
S8	Flamenco_R4-9	S56	Black Magic
S9	FRED_R10-4	S57	3J
S10	McL-Pand_R10-3	S58	Lacey
S11	MG_Griff_R15-7	S59	No. 10
S12	MG_Smith_R10-18	S60	AV-1
S13	MG_Smith_R10-19	S61	96A
S14	MG_Smith_R4-11	S62	McLeods Pandora
S15	MG_Smith_R4-12	S63	Tweed Pearl
S16	MGA_R9-16	S64	C18V9
S17	MGB_R4-4	S65	McGuffies_Samba
S18	MGC_R4-18	S66	BCF1-17
S19	No12_R5-4	S67	Black Magic_Bunnings
S20	No27_R4-1	S68	Tweed Tango
S21	P12-16-33_R8-19	S69	Misty Gem
S22	P12-16-37_R8-18	S70	Tweed Gem
S23	RG_R19-7	S71	Sweetheart
S24	SH_R10-6	S72	T x L_209
S25	SH_R10-7	S73	T x L_208
S26	SH_R4-7	S74	T x L_207
S27	SH_R4-8	S75	T x L_206
S28	SP-16-36D_R8-1	S76	T x L_205
S29	SP-16-36D_R8-13	S77	T x L_204

S30	SP-16-36D_R8-2	S78	T x L_203
S31	SP-16-59_R8-10	S79	T x L_202
S32	SP-16-59_R8-4	S80	T x L_201
S33	SP-16-90_R4-15	S81	Sunshine Splash
S34	SP-16-90_R8-8	S82	Jumbo Gem
S35	T12-16-4_R8-16	S83	McGuffie's Red
S36	T12-16-4_R9-5	S84	Bunnings Pandora
S37	TL-16-51_R4-2	S85	Tom's Special
S38	TL18-A68_R4-13	S86	20807_SH_PAI
S39	TL18-B5_R4-16	S87	T x L_210
S40	TomSp_R10-16	S88	Tweed Flamenco
S41	TomSp_R10-17	S89	Black Gem
S42	Z1_R6-1	S90	McGuffies_Samba2
S43	Z2_R6-2	S91	McGuffies
S44	Z3_R5-7	S92	23_E
S45	Z4_R10-12	S93	Pandora2015
S46	Z5_R9-12	S94	Panama Anderson
S47	SH_Qiagen	S95	Feral
S48	96A_R5_1		

Table S3. The 8 SSR markers used for the 95 scion accessions.

Marker ID	Forward (5′-3′)	Reverse (5′-3′)
PE27	TTGCTCATTGCACTCATCCT	GCAGACATTTCCTGGAGCA
mPs-16	GAGAAAGCGAGTCAGCGAGA	GACTCCAATATCGGCACTTCA
mPe-02	TCGAGTGAGATTGGCAGTG	TTGGCTTCGAGGAGAAGAA
PE-09	GGAAATCCGAAAACTGGTTG	GGGCCTTTATCCATGTTTGA
AG-12996	ATGTCCCTCTACTCTGATCC	TGAAGTTTGGAATCTCCCTG
mPs-01	TAGCTTAACACAATGCAACAGA	CAACGGAGAACGATGTCAG
USP-18	CCGTGAACCAACCATTTCTC	TTGCAGCACAAACAAGTCAA
PE26	GCTTTTCATATTTCGGGTTG	TTGCTTGAGTTTGGAGGAAG

Table S4. The average membership proportion of rootstock accessions in the structure analysis.

ID	POP1	POP2	POP3	POP4	POP5	POP6
17-001_A	0	0	0.0016	0.9954	0.0030	0
17-001_B	0.0002	0	0.0008	0.9964	0.0022	0
17-001_C	0.0002	0	0.0016	0.9964	0.0020	0
17-001_D	0	0	0.0008	0.9962	0.0026	0
17-002_A	0	0	0	0.0010	0.9982	0
17-002_B	0	0	0	0.0020	0.9980	0
17-002_C	0.0002	0	0.0006	0.0020	0.9972	0
17-002_D	0	0	0.0006	0.0026	0.9970	0
17-003_B	0	0	0.0006	0.4890	0.5102	0
17-003_C	0	0	0.0014	0.4860	0.5124	0
17-003_D	0	0	0.0006	0.4860	0.5134	0

17-004_A	0	0	0.0016	0.4920	0.5070	0
17-004_B	0	0	0.0006	0.4930	0.5068	0
17-004_C	0	0	0.0006	0.4920	0.5074	0
17-004_D	0	0	0.0008	0.4920	0.5072	0
17-005_A	0	0	0.0006	0.5208	0.4790	0
17-005_B	0	0	0.0016	0.5228	0.4760	0
17-005_C	0	0	0.0056	0.5210	0.4732	0
17-005_D	0.0002	0	0.0016	0.5230	0.4752	0
17-007_A	0.0002	0	0.4956	0.5034	0.0006	0
17-007_B	0	0	0.4956	0.5020	0.0020	0
17-007_C	0.0002	0	0.0016	0.9956	0.0030	0
17-007_D	0	0	0.0022	0.9480	0.0500	0
17-008_A	0	0	0.9950	0.0030	0.0022	0
17-008_B	0	0.0008	0.9982	0.0008	0	0
17-008_C	0	0	0.9988	0.0006	0.0002	0
17-008_D	0.0002	0.0016	0.9602	0.0306	0.0072	0
17-009_A	0	0.0012	0.0006	0.5068	0.4914	0
17-009_B	0	0.0020	0	0.9970	0.0002	0
17-009_C	0.0002	0.0050	0.0356	0.9316	0.0274	0
17-009_D	0	0.0016	0	0.9980	0	0.0004
17-010_A	0.0004	0.0004	0.5402	0	0.4590	0
17-011_A	0.0002	0	0.0006	0	0.9988	0
17-011_B	0	0.0010	0.0006	0	0.9970	0.0014
17-011_C	0.0002	0.0008	0.0006	0	0.9984	0
17-011_D	0.0002	0.0010	0.0006	0.0004	0.9976	0
17-012_A	0	0.0002	0.5008	0	0.4986	0
17-012_B	0.0010	0.0010	0.4976	0.0016	0.4990	0
17-012_C	0.0002	0	0.5006	0	0.4990	0
17-012_D	0.0002	0.0008	0.4936	0.0004	0.5050	0
17-016_A	0.0008	0	0.5356	0.0004	0.4634	0
17-016_B	0.0002	0.0018	0.5410	0	0.4566	0
17-016_C	0	0	0.5368	0	0.4628	0
17-016_D	0.0002	0.0022	0.0012	0	0.9964	0
17-017_A	0	0.0016	0.0006	0.5230	0.4750	0
17-017_B	0.0002	0.0020	0.0006	0.5230	0.4740	0.0002
17-017_C	0.0002	0	0.0006	0.5230	0.4762	0
17-017_D	0.0002	0.0006	0.0006	0.5200	0.4790	0
17-018_A	0	0.0010	0.0006	0.4960	0.5020	0
17-018_B	0.0002	0.0028	0.0006	0.4920	0.5044	0
17-018_C	0	0.0002	0	0.4900	0.5092	0.0006
17-018_D	0	0.0228	0	0.4822	0.4950	0
17-019_A	0	0.0134	0.9866	0	0	0
17-019_B	0.2824	0.1056	0.5106	0.0004	0.1010	0
17-019_C	0.0002	0.0040	0.9958	0	0.0002	0

17-026_A	0.7980	0	0.2020	0	0	0
17-026_C	0.9006	0	0.0994	0	0	0
17-026_D	0.7472	0	0.2422	0	0.0108	0
17-027_A	0.0296	0.0368	0.6574	0.0402	0.2362	0
17-029_A	0	0.0012	0.0004	0	0.9972	0.0008
17-029_B	0	0.0012	0	0	0.9980	0.0006
17-029_C	0.0002	0.0008	0.0004	0	0.9984	0
17-029_D	0	0.0020	0.0006	0	0.9970	0
17-264_A	0	0.0016	0.0002	0.0002	0.9980	0
17-264_B	0	0.0016	0	0	0.9974	0.0004
17-264_C	0	0.0018	0.0006	0	0.9972	0
17-264_D	0.0002	0.0016	0.0006	0.0002	0.9970	0
17-265_A	0.0002	0	0.4976	0.5020	0.0002	0
17-265_B	0.0002	0.0008	0.4946	0.5042	0	0.0004
17-265_C	0.0002	0.0016	0.4960	0.5020	0	0.0002
17-265_D	0	0.0012	0.9986	0	0	0
17-266_A	0.0002	0	0.4980	0	0.5016	0
17-266_B	0.0002	0	0.4990	0	0.5008	0
17-266_C	0.0002	0.0004	0.5010	0	0.4986	0
17-266_D	0.0002	0.0004	0.4980	0	0.5012	0
17-267_A	0.0002	0.0018	0.0022	0	0.9952	0.0002
17-267_B	0	0.0008	0.0006	0.0014	0.9970	0
17-267_C	0.0002	0.0026	0.0046	0	0.9926	0
17-267_D	0.0002	0.0024	0.0006	0	0.9968	0.0002
17-268_A	0.0002	0.0028	0.0006	0	0.9962	0
17-268_B	0.0002	0.0024	0	0	0.9970	0
17-268_C	0	0.0060	0	0	0.9934	0
17-268_D	0	0.0008	0.0004	0	0.9984	0
17-269_A	0	0.0030	0	0	0.9970	0
DurGrifPand	0	0.0008	0	0	0.9980	0.0002
Dur_Bun_Pan	0	0.0016	0.0006	0.0002	0.9976	0.0002
Dur_McL_Pan	0	0.0028	0	0	0.9970	0.0002
SP16-22D	0	0	0	0	0.0010	0.9984
SP16-36D	0	0.0052	0	0	0.0566	0.9380
SP16-42D	0.0002	0.0032	0.0006	0	0.9956	0
SP16-6	0	0	0	0	0.0070	0.9930
SP16-78	0	0	0	0	0.0052	0.9946
SP16-90	0	0	0	0	0.0004	0.9996
SP16-91	0	0	0	0	0.0732	0.9264

Table S5. The average membership proportion of scion accessions in the structure analysis.

ID	POP1	POP2	ID	POP1	POP2
295_R10-15	0.9948	0.0052	96A_R5_1	0.9925	0.0075
295_R10-5	0.9960	0.0040	96A_R5_2	0.9927	0.0073
295_R10-8	0.9960	0.0040	96A_No12_R4_14	0.9906	0.0094
296_R10-10	0.9960	0.0040	96A_No12_R4_15	0.9903	0.0097
302_R10-2	0.9942	0.0058	96A_No12_R6_8	0.9923	0.0077
AG27_R5-10	0.9969	0.0031	96A_No12_R6_15	0.9928	0.0072
AT-1_R17-8	0.9960	0.0040	DB_1_R5_16	0.9963	0.0037
Flamenco_R4-9	0.9938	0.0062	DPI	0.0030	0.9970
FRED_R10-4	0.9574	0.0426	Black Magic	0.0050	0.9950
McL-Pand_R10-3	0.9921	0.0079	3J	0.0080	0.9920
MG_Griff_R15-7	0.9960	0.0040	Lacey	0.0055	0.9945
MG_Smith_R10-18	0.9960	0.0040	No. 10	0.0070	0.9930
MG_Smith_R10-19	0.9950	0.0050	AV-1	0.0057	0.9943
MG_Smith_R4-11	0.9949	0.0051	96A	0.0058	0.9942
MG_Smith_R4-12	0.9952	0.0048	McLeods Pandora	0.0040	0.9960
MGA_R9-16	0.9967	0.0033	Tweed Pearl	0.0050	0.9950
MGB_R4-4	0.9950	0.0050	C18V9	0.0225	0.9775
MGC_R4-18	0.9960	0.0040	McGuffies_Samba	0.0031	0.9969
No12_R5-4	0.9970	0.0030	BCF1-17	0.0030	0.9970
No27_R4-1	0.9961	0.0039	Black Magic_Bunnings	0.0084	0.9916
P12-16-33_R8-19	0.9950	0.0050	Tweed Tango	0.0084	0.9916
P12-16-37_R8-18	0.9950	0.0050	Misty Gem	0.0076	0.9924
RG_R19-7	0.9951	0.0049	Tweed Gem	0.0055	0.9945
SH_R10-6	0.9970	0.0030	Sweetheart	0.0065	0.9935
SH_R10-7	0.9970	0.0030	T x L_209	0.0050	0.9950
SH_R4-7	0.9970	0.0030	T x L_208	0.0048	0.9952
SH_R4-8	0.9970	0.0030	T x L_207	0.0040	0.9960
SP-16-36D_R8-1	0.9950	0.0050	T x L_206	0.0057	0.9943
SP-16-36D_R8-13	0.9950	0.0050	T x L_205	0.0043	0.9957
SP-16-36D_R8-2	0.9960	0.0040	T x L_204	0.0049	0.9951
SP-16-59_R8-10	0.9950	0.0050	T x L_203	0.0040	0.9960
SP-16-59_R8-4	0.9950	0.0050	T x L_202	0.0034	0.9966
SP-16-90_R4-15	0.9955	0.0045	T x L_201	0.0054	0.9946
SP-16-90_R8-8	0.9949	0.0051	Sunshine Splash	0.0043	0.9957
T12-16-4_R8-16	0.9959	0.0041	Jumbo Gem	0.0050	0.9950
T12-16-4_R9-5	0.9950	0.0050	McGuffie's Red	0.0040	0.9960
TL-16-51_R4-2	0.9952	0.0048	Bunnings Pandora	0.0050	0.9950
TL18-A68_R4-13	0.9721	0.0279	Tom's Special	0.0062	0.9938
TL18-B5_R4-16	0.9958	0.0042	20807_SH_PAI	0.0075	0.9925
TomSp_R10-16	0.9950	0.0050	T x L_210	0.0062	0.9938
TomSp_R10-17	0.9948	0.0052	Tweed Flamenco	0.0060	0.9940

Z1_R6-1	0.9950	0.0050	Black Gem	0.0056	0.9944
Z2_R6-2	0.9933	0.0067	McGuffies_Samba2	0.0040	0.9960
Z3_R5-7	0.9948	0.0052	McGuffies	0.0040	0.9960
Z4_R10-12	0.9834	0.0166	23_E	0.0062	0.9938
Z5_R9-12	0.9950	0.0050	Pandora2015	0.0035	0.9965
SH_Qiagen	0.9905	0.0095	Panama Anderson	0.0059	0.9941
Feral	0.0086	0.9914			

Table S6. The expected heterozygosity for each subpopulation in the structure analysis of the 95 scion accessions.

He	POP1	POP2
	0.480	0.585

Table S7. Individual heterozygosity values of the 95 scion accessions based on SSR markers.

ID	PHt	Hs_obs	ID	PHt	Hs_obs
TomSp_R10-17	1.000	2.007	Sweetheart	0.500	1.004
Lacey	1.000	2.007	T x L_205	0.500	1.004
AV-1	1.000	2.007	T x L_201	0.500	1.004
Tweed Pearl	1.000	2.007	302_R10-2	0.375	0.753
Sunshine Splash	1.000	2.007	AG27_R5-10	0.375	0.753
Tom's Special	1.000	2.007	FRED_R10-4	0.375	0.753
Tweed Flamenco	1.000	2.007	MGB_R4-4	0.375	0.753
Black Gem	1.000	2.007	P12-16-37_R8-18	0.375	0.753
23_E	1.000	2.007	SP-16-36D_R8-1	0.375	0.753
Flamenco_R4-9	0.875	1.756	SP-16-36D_R8-13	0.375	0.753
MG_Smith_R4-12	0.875	1.756	SP-16-59_R8-10	0.375	0.753
96A	0.875	1.756	SP-16-59_R8-4	0.375	0.753
Misty Gem	0.875	1.756	T12-16-4_R9-5	0.375	0.753
T x L_208	0.875	1.756	TL18-B5_R4-16	0.375	0.753
T x L_202	0.875	1.756	Z1_R6-1	0.375	0.753
MG_Smith_R10-19	0.750	1.506	DPI	0.375	0.753
MG_Smith_R4-11	0.750	1.506	McLeods Pandora	0.375	0.753
TomSp_R10-16	0.750	1.506	T x L_206	0.375	0.753
96A_R5_1	0.750	1.506	Pandora2015	0.375	0.753
96A_R5_2	0.750	1.506	MGA_R9-16	0.250	0.502
96A_No12_R6_8	0.750	1.506	MGC_R4-18	0.250	0.502
96A_No12_R6_15	0.750	1.506	No12_R5-4	0.250	0.502
Black Magic	0.750	1.506	No27_R4-1	0.250	0.502
C18V9	0.750	1.506	P12-16-33_R8-19	0.250	0.502
T x L_209	0.750	1.506	SH_R10-6	0.250	0.502
T x L_203	0.750	1.506	SH_R10-7	0.250	0.502
Jumbo Gem	0.750	1.506	SH_R4-7	0.250	0.502
T x L_210	0.750	1.506	SH_R4-8	0.250	0.502
MG_Griff_R15-7	0.625	1.255	TL-16-51_R4-2	0.250	0.502
MG_Smith_R10-18	0.625	1.255	SH_Qiagen	0.250	0.502

RG_R19-7	0.625	1.255	96A_No12_R4_14	0.250	0.502
Z2_R6-2	0.625	1.255	96A_No12_R4_15	0.250	0.502
Z3_R5-7	0.625	1.255	McGuffies_Samba	0.250	0.502
Z4_R10-12	0.625	1.255	295_R10-15	0.125	0.251
Z5_R9-12	0.625	1.255	295_R10-5	0.125	0.251
3J	0.625	1.255	295_R10-8	0.125	0.251
No. 10	0.625	1.255	McGuffies	0.125	0.251
Tweed Gem	0.625	1.255	296_R10-10	0.000	0.000
T x L_207	0.625	1.255	AT-1_R17-8	0.000	0.000
T x L_204	0.625	1.255	McL-Pand_R10-3	0.000	0.000
20807_SH_PAI	0.625	1.255	DB_1_R5_16	0.000	0.000
SP-16-36D_R8-2	0.500	1.004	Black Magic_Bunnings	0.000	0.000
SP-16-90_R4-15	0.500	1.004	McGuffie's Red	0.000	0.000
SP-16-90_R8-8	0.500	1.004	Bunnings Pandora	0.000	0.000
T12-16-4_R8-16	0.500	1.004	McGuffies_Samba2	0.000	0.000
TL18-A68_R4-13	0.500	1.004	Panama Anderson	0.000	0.000
BCF1-17	0.500	1.004	Feral	0.000	0.000
Tweed Tango	0.500	1.004			

Table S8. Individual heterozygosity values of the 94 rootstock accessions based on SNP markers.

ID	PHt	Hs_obs	ID	PHt	Hs_obs
SP16-22D	0.462	3.041	17-026C	0.058	0.380
SP16-90	0.455	2.994	17-009C	0.054	0.354
SP16-78	0.451	2.968	17-007D	0.051	0.336
SP16-36D	0.449	2.953	17-264B	0.047	0.306
SP16-6	0.444	2.920	17-264A	0.046	0.305
SP16-91	0.436	2.868	17-267C	0.046	0.300
17-004C	0.319	2.096	17-267B	0.045	0.298
17-004D	0.318	2.095	17-267D	0.045	0.298
17-004A	0.318	2.091	17-267A	0.045	0.293
17-004B	0.317	2.088	17-264D	0.044	0.289
17-003C	0.314	2.067	17-264C	0.044	0.289
17-003D	0.314	2.064	17-268B	0.043	0.282
17-003B	0.312	2.055	17-008D	0.042	0.275
17-018B	0.303	1.992	17-268D	0.039	0.259
17-018A	0.301	1.983	17-269A	0.033	0.216
17-018C	0.300	1.976	17-027A	0.032	0.212
17-005A	0.297	1.957	17-268A	0.032	0.210
17-009A	0.297	1.953	17-011B	0.030	0.198
17-005C	0.296	1.951	17-029D	0.029	0.191
17-005B	0.296	1.950	17-011C	0.029	0.190
17-005D	0.296	1.945	Sweetheart	0.027	0.178
17-017B	0.287	1.887	17-008A	0.027	0.175
17-017A	0.286	1.882	17-029B	0.026	0.169

17-017D	0.285	1.876	17-265D	0.026	0.168
17-017C	0.283	1.861	17-019A	0.025	0.167
17-007B	0.241	1.583	17-008B	0.025	0.167
17-007A	0.240	1.582	17-008C	0.025	0.164
17-266A	0.237	1.556	17-001D	0.025	0.164
17-266D	0.236	1.552	17-001C	0.025	0.164
17-266C	0.235	1.548	17-009B	0.025	0.162
17-012B	0.235	1.543	17-029C	0.024	0.156
17-266B	0.233	1.534	DurMcLPandora	0.023	0.152
17-265C	0.232	1.528	17-007C	0.023	0.151
17-012A	0.232	1.527	17-029A	0.023	0.151
17-012C	0.232	1.525	17-001A	0.023	0.151
17-265B	0.232	1.525	17-001B	0.023	0.148
17-012D	0.231	1.522	DurGrifPandora	0.023	0.148
17-265A	0.230	1.514	17-002A	0.022	0.147
17-010A	0.216	1.421	17-009D	0.022	0.146
17-016A	0.215	1.416	17-002B	0.022	0.144
17-016C	0.214	1.406	DurBunPandora	0.022	0.144
17-016B	0.212	1.397	17-011A	0.021	0.141
17-018D	0.187	1.233	17-016D	0.021	0.140
17-019B	0.137	0.900	17-002D	0.021	0.138
17-026A	0.076	0.500	17-268C	0.021	0.137
17-026D	0.065	0.425	17-002C	0.020	0.134
SP16-22D	0.462	3.041	17-011D	0.020	0.133

Table S9. Dissimilarity results among rootstock accessions based on DArTseq SNP markers. (See SUN_Supplementary2_MDPI_20250322.csv)

Table S10. The PIC values of the eight SSR markers used in this study.

**Locus**	**PIC**
PE27	0.602
mPs-16	0.339
mPe-02	0.587
PE-09	0.393
AG-12996	0.704
mPs-01	0.450
USP-18	0.698
PE26	0.582

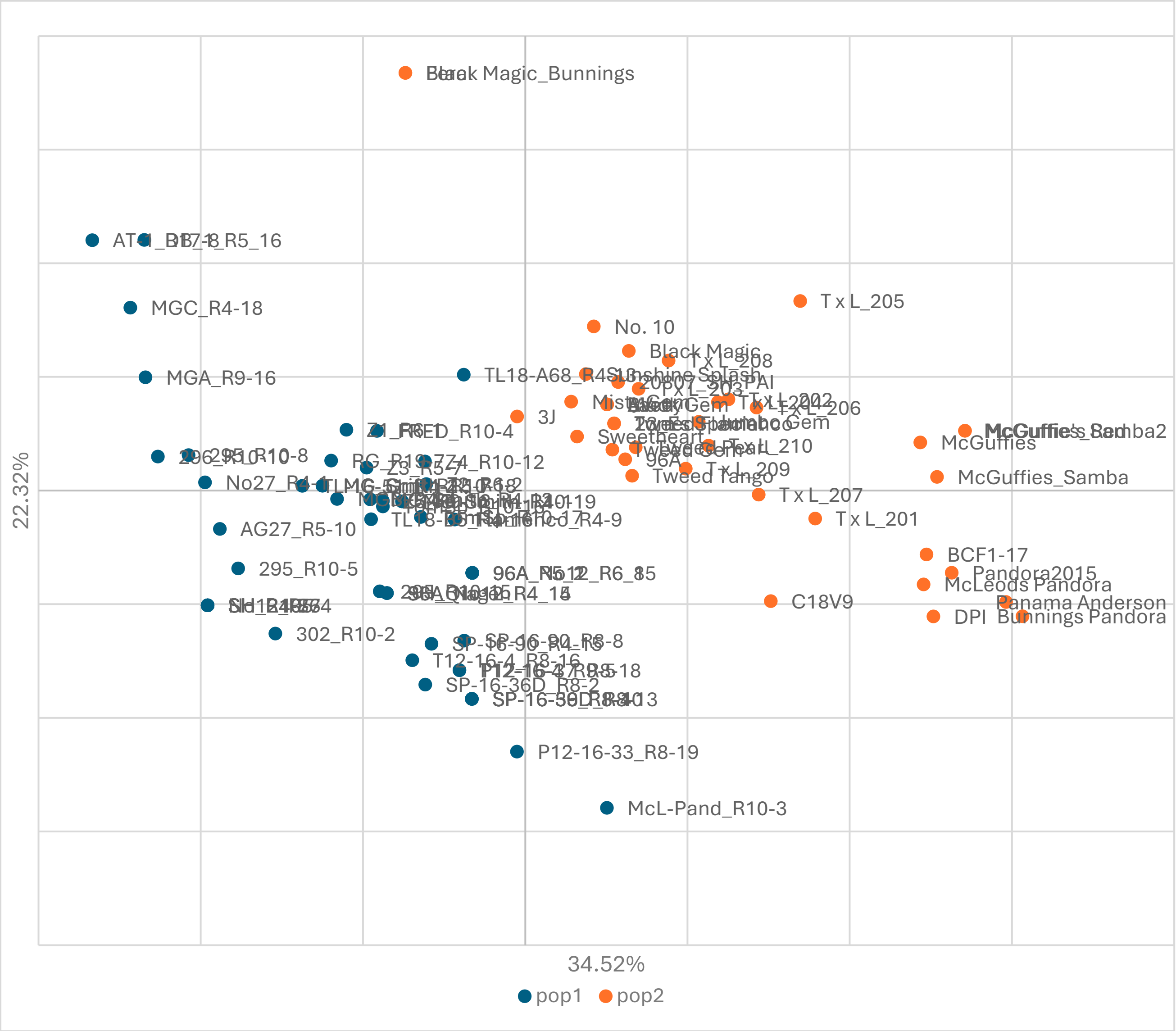

Figure S1. Principal coordinate analysis (PCoA) of the 95 scion accessions.

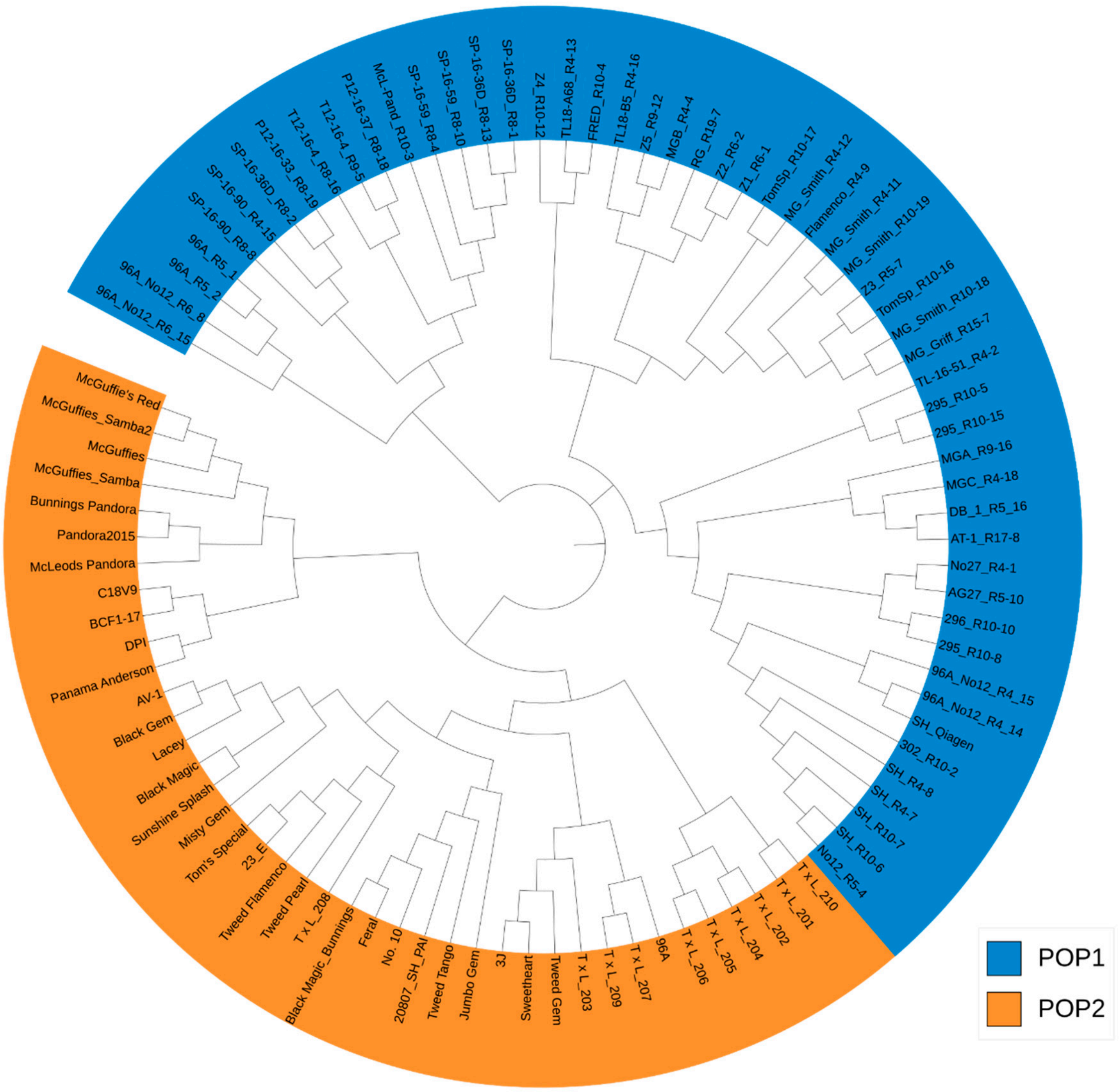

Figure S2. Weighted neighbour-joining dendrogram of the 95 scion accessions.
